# Diseases of Colliery Operatives, and Their Preventible Causes

**Published:** 1857-01

**Authors:** William I. Cox

**Affiliations:** Corresponding Member of the Epidemiological Society.


### ON THE DISEASES OF COLLIERY OPERATIVES; AND THEIR PREVENTIBLE CAUSES.

By WILLIAM I. COX, M,R.C.S., etc., Corresponding Member of the Epidemiological Society.

(*Continued from* p. 43.)

In the former part of this paper, I have spoken only of the *actual diseases* to which colliery operatives are rendered liable by the nature of their employment; and the *specific causes*, as far as these are readily traceable, of the maladies arising out of their mode of labour and habits of life. But, before I proceed to the suggestion and detail of measures directed either towards the prevention and removal of such causes, or where this is not possible or practicable, their neutralisation or mitigation; it will not be out of place or foreign to the subject to devote a brief consideration to several additional circumstances, connected with the daily career of this class of operatives, over which they themselves possess little or no control—conditions and influences, universally acknowledged to be potent agents of evil; which are necessarily associated with the nature of the employment in which they are engaged, and which they are generally quite powerless to alter or avoid. Hence, the study of the really preventible causes of disease among colliers, becomes properly invested with a graver importance and a weightier claim to our interest, when it is borne in mind that there are many injurious influences at work, which we in truth *cannot avert;* circumstances which we cannot change, but which gradually impair the bodily powers, and are, as a necessary consequence, powerfully predisponent to the inroads of the maladies of which I have already spoken. It is surely fair to say that, if a man be, by the nature of his daily labour and the circumstances surrounding him, subject continually and inevitably to the operation and influence of agents unfavourable to health, it behoves him to be doubly careful to shun those causes effecting the diminution of the vital energies and the impairment of the bodily functions which he *has* the power to avoid. That these unavoidable predisposing causes of disease and direct producers of debility and general loss of tone, are by no means slight, unimportant or partial in their ultimate results, may be inferred from the indisputable fact of the early decline and mortality of colliery operatives. Even among those who otherwise lead a regular life, and are not guilty of any systematic voluntary acts tending to shorten life, premature old age is generally apparent at the age of forty-five; and life, indeed, is rarely prolonged after sixty. It is unquestionable, that instances of longevity among pit-men are very uncommon. The characteristics of this early, and it would appear unavoidable decay and decline, will presently be more particularly described.

Of the principle of these hurtful influences, I shall first mention,

1. *Privation of Solar Light.* It is of course quite unnecessary to fill up the valuable space of this journal by any physiological argument to prove, or examples to illustrate the power, which the sun's rays (direct and refracted) possess for the maintenance of health, the prevention of disease, and also for the removal of the latter and the restoration to an integral state. The privation of solar light exercises an effect on man, precisely analogous to that which it produces on the vegetable kingdom—differing only in degree. The *plant* from which the light is excluded grows up sickly, pale, watery—its leaves nearly destitute of their natural flavour—its stalk fragile and brittle—its proper juices either perverted in nature or deficiently elaborated. *Man*, under similar circumstances, becomes also pale; and, as it were, etiolated—his complexion blanched and sallow—his limbs stunted and generally somewhat curved—his countenance heavy and listless in its expression—his blood poor, watery, deficient in albuminous constituents and in *red* corpuscles, whilst the proportion of *colourless* discs is increased (leucocythæmia, or white-cell blood). In short, the protracted exclusion of light results in a condition of vital depression, attended with increased sensibility and susceptibility to impressions—indeed, in a spanæmic state, and a general and somewhat peculiar cachexia. It must be evident to the most superficial observer, that light—especially sun-shine—is ordained by nature as a salutary stimulus, and necessary to the full and healthy performance of all the bodily functions: and that its partial abstraction for a lengthened period must be ultimately followed, if not by actual disease, at least by a systemic condition very far below par, and having a decided proclivity to various maladies.

Now, of all classes of labourers, colliers and miners are especially liable to the injurious effects of the privation of solar light; whatever these may be. During the winter months (November, December, January, and February) it may be said that they seldom see daylight; as they generally descend the pits at about four or five, a.m., not returning to the surface until the same hour p.m. And during the period of longer days and brighter weather, they are rarely above ground longer, at most, than three or four hours before sunset. Thus, the whole year round, they enjoy very little of the light of day, and still less sunshine; excepting, of course, on the Sabbath and pay-days. And that they are cut off from the beneficial influences of sunlight and pure wholesome air, is sufficiently evidenced, not only in the prevalence among them of the diseases to which they are especially liable, but also in the bleached, sallow, unhealthy countenances, which (in accordance with the general principles above described) they present, when the extraneous dirt, with which their skin is usually loaded, is removed.

2. *The unnatural atmosphere* in which these men are compelled to labour, may perhaps be justly accounted the next fertile and inevitable source of bodily mischief, and lowering of the energies of life, to which they are subjected. This injurious atmosphere generally embraces four or five conditions, all very detrimental to health, viz. :—

*a.* An insufficient supply of pure air.
*b.* The presence of noxious gases.
*c.* The presence of mechanical impurities.
*d.* Excessive moisture.
*e.* Sudden and violent changes in the condition of the air.

All these conditions and circumstances of the atmospheric medium have a direct tendency to one result, viz., interference with the proper performance of the respiratory function; and, as a necessary consequence, with the depurating processes, whereby the lungs and liver preserve the purity and health of the circulating fluid and its derivatives. Frequently these conditions are all present together; and combine to produce, by their persistence in the course of time, an universal cachexia; and to lay the foundation of, and highly predispose the frame to, structural maladies of the lungs, heart, liver, and kidneys.

*a.* The ill effects of arts and trades, conducted in close and badly ventilated chambers, is too well known to require comment. The next condition (*b*) is, however, necessarily associated with it, in so far as respects the admixture of carbonic acid gas.

*b.* The deleterious admixture of gases, foreign to the constitution of the atmosphere, is a very serious and highly important evil. These noxious agents, as they exist in coal mines, consist chiefly of light carbide of hydrogen (fire-damp, $C\,H_2$); carbonic acid; sometimes sulphurous acid gas (from the pit-fires); and free hydrogen (very rare). The effect of the frequent presence of these gases in the air, is to interfere with the proper transpiration of carbonic acid gas from the pulmonary membrane; and its consequent accumulation in the blood—to which the prevalent disorders of the cerebral circulation and organs of vision bear testimony. The particular mischievous effects of the respiration of even a minute percentage of carbonic acid in the atmosphere, are as well known at the present day (or at all events *should* be) to the general, as to the professional reader.

*c.* The peculiar mischief induced in the pulmonary tissue by the *inhalation of coal-dust* and other carbonaceous matters in minute solid particles, has already been described at length. (See page 40.)

*d.* The evil consequences of long-continued dwelling in a preternaturally *moist atmosphere,* are not quite so obvious as those already referred to. There can be small doubt, however, but that it *is* productive of much mischief, and tends, by the low temperature which it must necessarily occasion, to depress the energies, produce a species of stagnation, and seriously impede various bodily functions—particularly those of secretion and excretion.

*e.* The influence of abrupt and decided *changes in the chemical condition of the atmosphere,* and of *variations of temperature,* when of frequent occurrence, must ultimately be highly prejudicial to those persons subjected to them; especially when, as is commonly the case, there is utter neglect of all precaution calculated to enfeeble or neutralise their effects. The sudden shocks thus caused, affecting the delicate machinery of the air-cells, often repeated, result finally in structural lesion. And in this manner the basis is slowly, but surely, laid of the worst form of asthma (emphysematous lung); as well as of those maladies of the circulation, which follow in its train. The effects of the same transitions and changes are perhaps no less hurtful, acting as they also do through the medium of the cutaneous surface; favouring internal congestion; and quickening the development of local morbid processes in the chest and abdomen.

3. *The constrained and unpleasant postures* in which colliers are obliged to work, can scarcely fail to lead to some degree of bodily deformity, and proneness to functional and even structural derangement of some of the viscera. They are frequently compelled to labour for hours in a sitting or lying posture; and occasionally also in a cramped and painful position, in which some particular muscles are kept in a prolonged state of unnatural tension. Mention has previously been made (in my former paper) of the great probability there exists of this state of things resulting sometimes in renal, sometimes in cardiac, disease. But its effect is more commonly and manifestly apparent in the outward conformation of colliers, and in the well known fact that the majority of them (those at least who have been trained to pit-labour from their early youth) are more or less *bow-legged;* and that large numbers of them have also slight curvature of the spine.

I may also remark, that colliery operatives are often under the necessity of pursuing their labour in localities where the water constantly reaches above the ankle—and this sometimes, day after day, for many weeks in succession. Can we, then, be surprised at the prevalence of rheumatic arthritis, bronchitis, and pleuritis, amongst men working under such unfavourable circumstances?

(*To be concluded.*)

---

